# Prototyping and evaluation of a clinical decision support system in long-term care in German nursing homes

**DOI:** 10.1007/s10729-026-09794-2

**Published:** 2026-10-10

**Authors:** Maximilian Pilz, Anne Gebert, Matthias Brünett, Laura Steffny, Christina Schubert, Jonas Flechsig, Alexander Scherrer

**Affiliations:** 1 Fraunhofer Institute for Industrial Mathematics, Kaiserslautern, Germany; 2 August-Wilhelm Scheer Institute for Digital Products and Processes gGmbH, Saarbrücken, Germany; 3 German Institute for Applied Nursing Research, Cologne, Germany

**Keywords:** Long-term care, Clinical decision support system, Large language model, Nursing intervention

## Abstract

Demographic change in Germany is increasing the demand for professional long-term care. Given rising requirements and simultaneous staff shortages, the need for digital systems is growing to effectively support nursing staff and mitigate staffing bottlenecks. This article introduces a hybrid clinical decision support system (CDSS) designed to generate actionable intervention recommendations by combining structured data and narrative nursing documentation. The CDSS consists of three interacting components: (1) knowledge models that encode state-of-the-art nursing knowledge, (2) a rule-based expert system that applies these models to generate individualized nursing interventions within the application fields mobility and pain management, and (3) a large language model (LLM) that extracts indicators that are necessary for knowledge-based decision-making from free-text entries to fill documentation gaps. A prototype was implemented and descriptively evaluated in two German nursing homes. Overall, 72% of the CDSS recommendations were rated as “correct” by nursing professionals, meaning appropriate for the care situation. In a separate evaluation, the LLM achieved 76% agreement rate in extracting care-relevant information from narratives. These results suggest that a hybrid architecture integrating expert knowledge, rule-based logic, and language models may have the potential to enhance nursing planning in long-term care settings.

## Highlights

- General approach to decision rules for nursing interventions based on expert knowledge – illustrated in two application areas: mobility and pain management.
- Demonstration of how language models can be used to extract decision-relevant information from free-text documentation.
- Evaluation of clinical decision support system for nursing interventions gives promising results.
- Current German nursing documentation system for long-term care shows gaps regarding relevant information for nursing interventions.

## Introduction

As in many other sectors, the potential roles of digital applications in the nursing sector are currently being explored. The nursing profession, especially in long-term care (LTC) in Germany, is facing challenges for which digital technologies could provide suitable tools and solutions. Germany, like other industrialized countries, is dealing with the implications of an aging population and a growing shortage of qualified personnel. Currently, 849,000 people in need of LTC are cared for in nursing homes in Germany. This number has increased over the last decade [1, 2] and is expected to increase to 1.1 million within the next 20 years [3]. At the same time, a shortage of approximately 300,000 skilled nurses is expected across all nursing domains in Germany by 2049 [3]. Conditions like multimorbidity are prevalent in up to 70% of older people in Germany [4], creating more complex care needs as well as leading to increased work intensification and more difficult conditions for care provision. Against the background of increasingly complex care needs, nursing documentation and care planning have become central components of professionally appropriate and patient-centered care. In this context, the German regulatory body for quality assurance in nursing homes (Medizinischer Dienst Bund – MD) plays a key role, as it routinely reviews nursing documentation and care plans [5], making individualized care planning a priority for nurses. To support nurses in addressing these increased demands and delivering individualized care, clinical decision support systems (CDSS) can offer valuable assistance.

Thompson et al. [6] define CDSS as “software- or computer-based technologies that offer patient-specific recommendations based on either research, expert opinion, machine learning/artificial intelligence or combinations of these, and designed to influence the clinical decision-making of health professionals.” In recent years, advances in artificial intelligence (AI), particularly in the field of large language models (LLMs), have broadened the capabilities of CDSS [7]. These advances have made it possible to extract crucial information from unstructured clinical text, interpret nursing documentation semantically, and integrate with existing expert systems [8]. CDSS thereby facilitate resource-efficient handling of complex planning situations with high-quality results [9, 10].

CDSS are typically categorized into knowledge-based and non-knowledge-based systems [11–13]. Knowledge-based systems depend on a set of established rules, whereas non-knowledge-based systems support decisions through data-driven approaches. These data-driven systems increasingly utilize machine learning, deep learning, and LLMs to generate predictions from clinical data [14]. However, this innovation brings several challenges, such as ensuring transparency and interpretability, mitigating biases, and complying with regulatory requirements [14].

To maximize transparency and reliability, we have developed a CDSS with a knowledge-based core that integrates a rule base grounded in nursing science evidence. Additionally, the system incorporates automatic structuring of unstructured data through an LLM, representing its non-knowledge-based component. To provide a solid foundation for decision-making, all parameters identified by the LLM were validated by practicing nursing professionals prior to further processing. Consequently, the developed CDSS can be regarded as a hybrid system with a strong emphasis on its knowledge-based component.

Different areas of application in nursing are covered by the CDSS. Some systems focus on specific aspects of nursing care, while others encompass multiple domains [15]. Examples of single areas include fall prevention, delirium care, body temperature monitoring, glucose level monitoring [15], palliative care [16], and pressure ulcers [17]. The output functions of CDSS also differ: Most systems provide recommendations and suggestions, followed by information management and monitoring, and alerts, notifications and reminders [18].

CDSS have the potential to improve the quality of care in different settings [11]. Numerous CDSS have already been developed for short-term use in clinical environments [19, 20]. In LTC settings, however, existing solutions address medication management or specific diseases but not the planning of nursing measures at admission [21]. Recent reviews consistently identify the gap of CDSS in LTC, specifically in the context of nursing homes [22–25]. With regard to technical capabilities, a recent review shows that the vast majority of available CDSS are knowledge-based systems, only one using machine learning [22], even though there is high potential for AI applications in CDSS [14]. Furthermore, most of the CDSS rely on nurses’ input; a minority can automatically retrieve data from nursing documentation or an electronic health record (EHR) [22].

The aim of the project presented in this paper was to develop and evaluate a prototype CDSS for the nursing home setting. The system is intended to support the admission process. Based on data from nursing documentation, the CDSS is intended to recommend appropriate nursing interventions in two exemplary areas of application: mobility and pain management. This paper presents the approach to developing the CDSS, along with the results of its evaluation. It is structured as follows: In Sect. 2, the relevant data sources for nursing home documentation in Germany are described. Section 3 introduces the methodology for developing a sound rule base for planning nursing interventions and how to use language models to extract relevant information from the free-text documentation. Section 4 presents the testing strategy and exploratory results of the evaluation in two care facilities. The paper concludes with a discussion in Sect. 5.

## Data sources in the nursing home setting in Germany

In Germany’s nursing home sector, providers typically use various data collection frameworks to support a range of organizational functions, including facilitating insurance benefits, care planning, and quality reporting [26]. As a result, the data landscape is characterized by semantic and conceptual heterogeneity and a variety of structured and unstructured data.

Information describing the residents’ health status and care needs is often documented as free-text, frequently using everyday language rather than professional terminology or standardized classifications. Medical diagnoses in the documentation do not necessarily comply with standardized classifications like ICD-10 [27], as physicians and hospitals often use non-coded, sometimes colloquial diagnostic entries. In contrast, structured data is routinely collected to assess eligibility for LTC insurance benefits and for quality assurance [26]. Medical prescriptions are also available as structured data complying to existing classifications [28]. Moreover, while relevant data is generally available in electronic form within the respective systems used by care providers, there is limited interoperability and very little systematic exchange of information between those systems.

Table 1 provides an overview of the organizational functions, associated tools, and the nature of data structures used in the admission process in German nursing homes.

**Table 1:** Overview of the most important data sources used in the admission process in nursing homes (Germany)

Primary Tool/ Source	Organizational functions	Data structure and format
Care Dependency Assessment (BI)	Administration (Determining the care dependency level for insurance benefits)	Structured assessment on defined ordinal scales
Structured Information Collection (SIS) including summary risk matrix	Care delivery (Assessment of care needs for care planning)	SIS: Primarily free-text fields and narrative documentation, with Summary risk matrix: Five defined topics and five care-sensitive risks
Medical diagnoses	Care delivery	Rarely coded (e.g., no ICD-10); typically descriptive and unstructured
Medical prescriptions	Care delivery	Structured information based on Central Pharmaceutical Number and Anatomical Therapeutic Chemical (ATC) Classification

The Care Dependency Assessment BI (Begutachtungsinstrument) [29] and the Structured Information Collection SIS (Strukturierte Informationssammlung) [30] will be explained in more detail below. Both instruments were specifically developed for and embedded in the German LTC setting and represent two key instruments and routinely available data sources in nursing homes.

### Care dependency assessment (BI)

The BI is the standardized tool for assessing care dependency and eligibility for insurance benefits in LTC. It identifies functional abilities across six domains: (1) mobility, (2) cognitive and communicative abilities, (3) behavior and psychological health, (4) self-care, (5) management of health-related needs, and (6) social participation. The BI includes approximately 70 items with over 250 defined response options for the standardized assessment of care dependency. The results determine the level of care dependency according to the German LTC insurance system, which is a prerequisite for accessing LTC insurance benefits [29]. The BI provides administrative information and is not used in direct patient care, for example as an assessment instrument.

### Structured information collection SIS

The SIS is part of a standard framework for nursing documentation that follows the WHO’s four-phase nursing process: Information Collection, Individualized Care Planning, Nursing Report, and Evaluation. As the central instrument to facilitate information collection, the SIS serves as a status assessment and is used to document relevant information for care planning. It is primarily used during admission and when significant changes in a resident’s condition occur. Any deviation from the documented baseline must be noted in the ongoing nursing report.

The SIS begins with the question “How are you?”, using a narrative approach to explore the individual's needs, experiences, and preferences before focusing on six thematic fields: (1) cognitive and communicative abilities, (2) mobility, (3) health-related conditions and burdens, (4) ability for self-care, (5) social relationships, and (6) living and housing. It uses a narrative approach to allow precise, individualized descriptions of residents’ functional abilities, needs, and risks. Free-text entries are mandatory; the use of pre-formulated text modules or templates is explicitly discouraged, a principle emphasized in official training materials. Additionally, a standardized risk matrix is used to assess key care risks regarding pressure ulcers, falls, incontinence, pain, and nutrition [30].

### Completeness of collected data and additionally introduced indicators

The goal of the developed CDSS is to make suggestions for nursing interventions based on nursing documentation. To determine which data is available or missing for decision support, we performed a preliminary analysis of 11 case data sets provided by the two nursing facilities involved in the research project. Relevant information for the application area mobility was drawn from the National Expert Standard “Maintaining and Promoting Mobility in Nursing Care” [25]. Relevant information for the application area pain was drawn from the Expert Standard “Pain Management in Nursing” [31] and the S3 Guideline: Pain Assessment in Older Adults in Long-Term Residential Care [32]. The German national expert standards provide evidence on objectives of nursing care and nursing interventions. They are mandatory and must be complied with by nursing care institutions. In the analysis, we matched information relevant for the two exemplary application areas mobility and pain management to the data sources of the nursing documentation BI, SIS, risk matrix, nursing reports, prescriptions/orders, and diagnoses. For pain management, we additionally included pain protocols and the initial pain assessments that are carried out upon residents’ admission, based on the clinical judgment of the nurse.

**Fig. 1:**
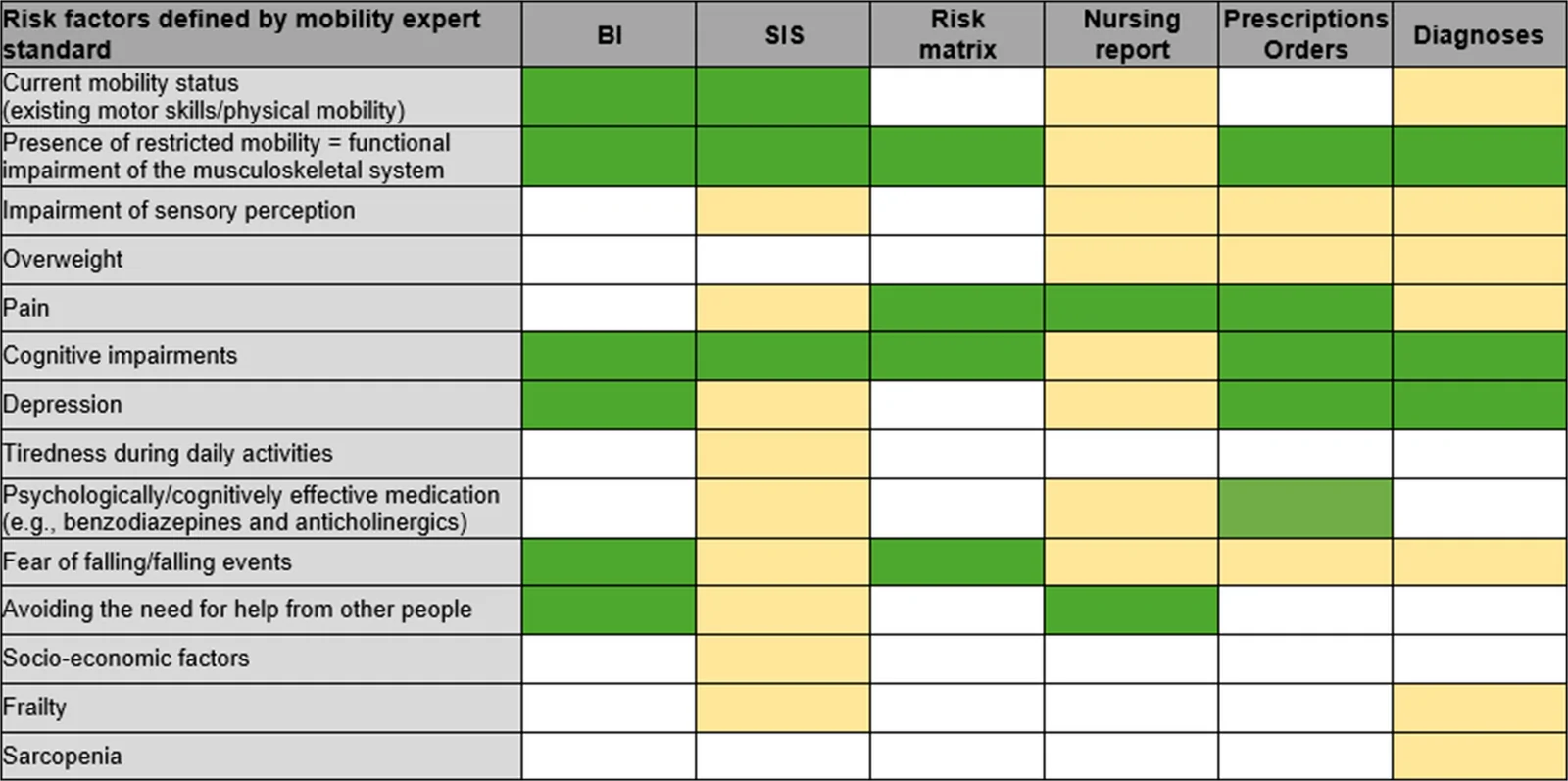
Availability of risk factors defined by mobility expert standard (green: consistently available, yellow: occasionally available, white: not available)

**Fig. 2:**
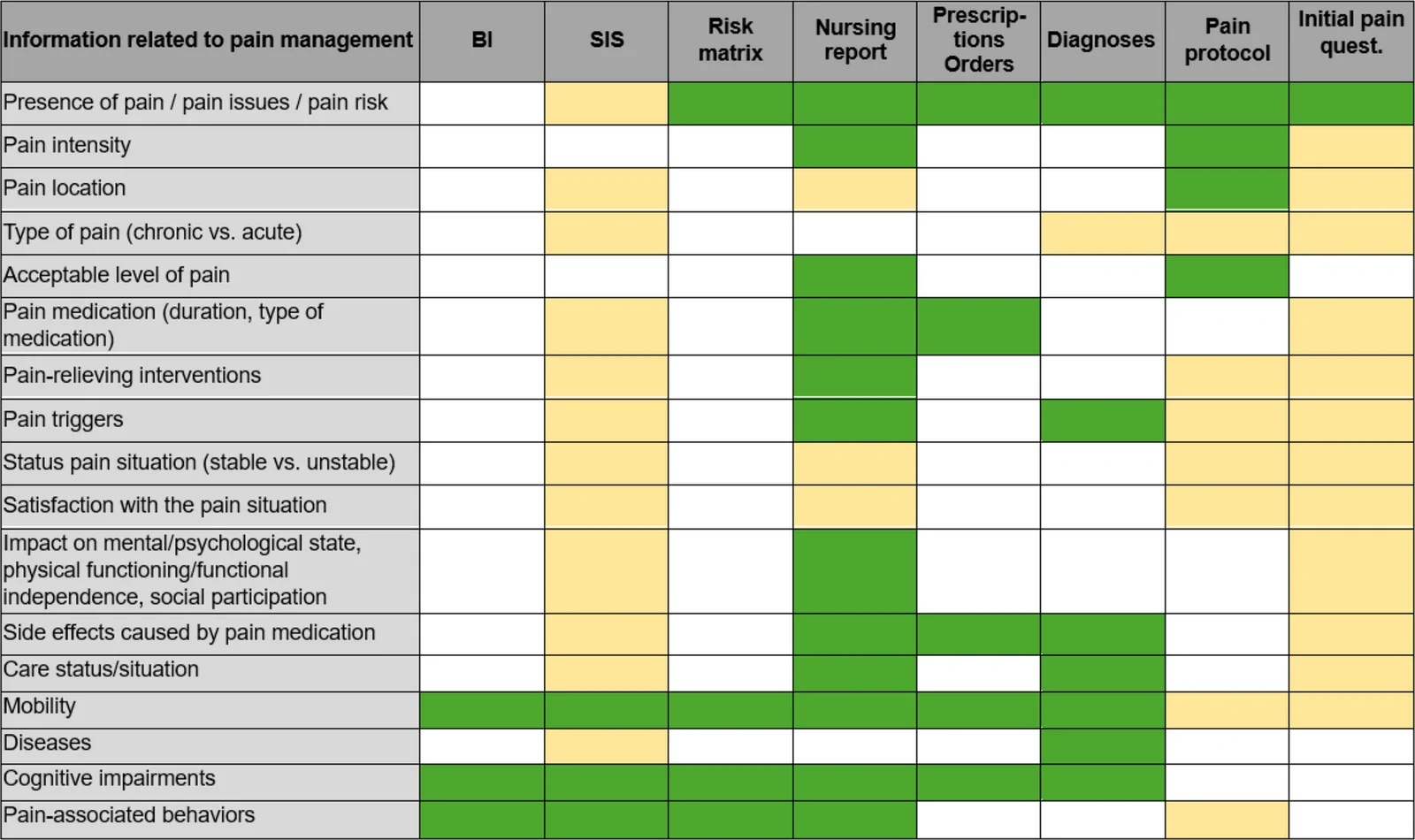
Availability of relevant information for pain management (green: consistently available, yellow: occasionally available, white: not available)

Figure 1 provides an overview of how complete mobility-related risk factors, as stated in the expert standard “Maintaining and Promoting Mobility in Nursing Care” [33], are available in routine nursing and medical documentation. Figure 2 provides an overview of the analyzed data relevant to pain management.

The results presented in Figs. 1 and 2 show that not all indicators identified as relevant for the application areas are available in the documentation. Only a few indicators are consistently available, most of them are only occasionally documented. This indicates inconsistent data availability on this topic. The reasons for these gaps remain unclear: the condition may be inapplicable to certain individuals, deemed insufficiently relevant, poorly addressed in practice, or simply not systematically documented. Additionally, relevant information is often scattered across different data sources. These findings indicate limited availability of several indicators in the documentation that are essential for intervention decisions in the areas of application. Furthermore, some of the consistent data was only available in an unstructured format.

Therefore, the following indicators were declared necessary for planning nursing interventions. These indicator collections were developed to ensure that all relevant information is collected in a compact and structured manner. For the area of application “mobility” 12 additional indicators are defined:

- **General medical history**: diagnosis dementia, diagnosis epilepsy, use of mobility aids, use of visual aids, history of falls, fear of falling,
- **Mobility and daily life indicators**: observable physical inactivity, daily routine without physical activity, observed decline in coordination, preference for low-activity routines, movement restricted to own room, receiving visits from relatives

For the application area “pain management” the nursing documentation lacked structured data, and data granularity was insufficient. Initial pain questionnaires and pain protocols are not standardized, resulting in variability in the assessed content. Moreover, their administration is at the discretion of nursing professionals. So, information on type of pain, pain location, and pain intensity were not systematically provided in the documentation and/or the granularity was too low to use it for nursing intervention recommendations. Information on self-reported pain coping mechanisms and perceived efficacy of various interventions, including both pharmacological and non-pharmacological treatments, is generally not recorded systematically. A lot of information is collected over time in the nursing report or progress notes which are not available upon residents’ admission and only documented in an unstructured manner. To address that issue, a questionnaire has been developed to facilitate data collection for testing the CDSS. The questionnaire includes a pain screening, a comprehensive pain assessment for information on the type of pain, pain intensity, pain localization, pain trigger, pharmacological and non-pharmacological treatment and self-reported pain management strategies.

## Description of clinical decision support system

### System overview

**Fig. 3:**
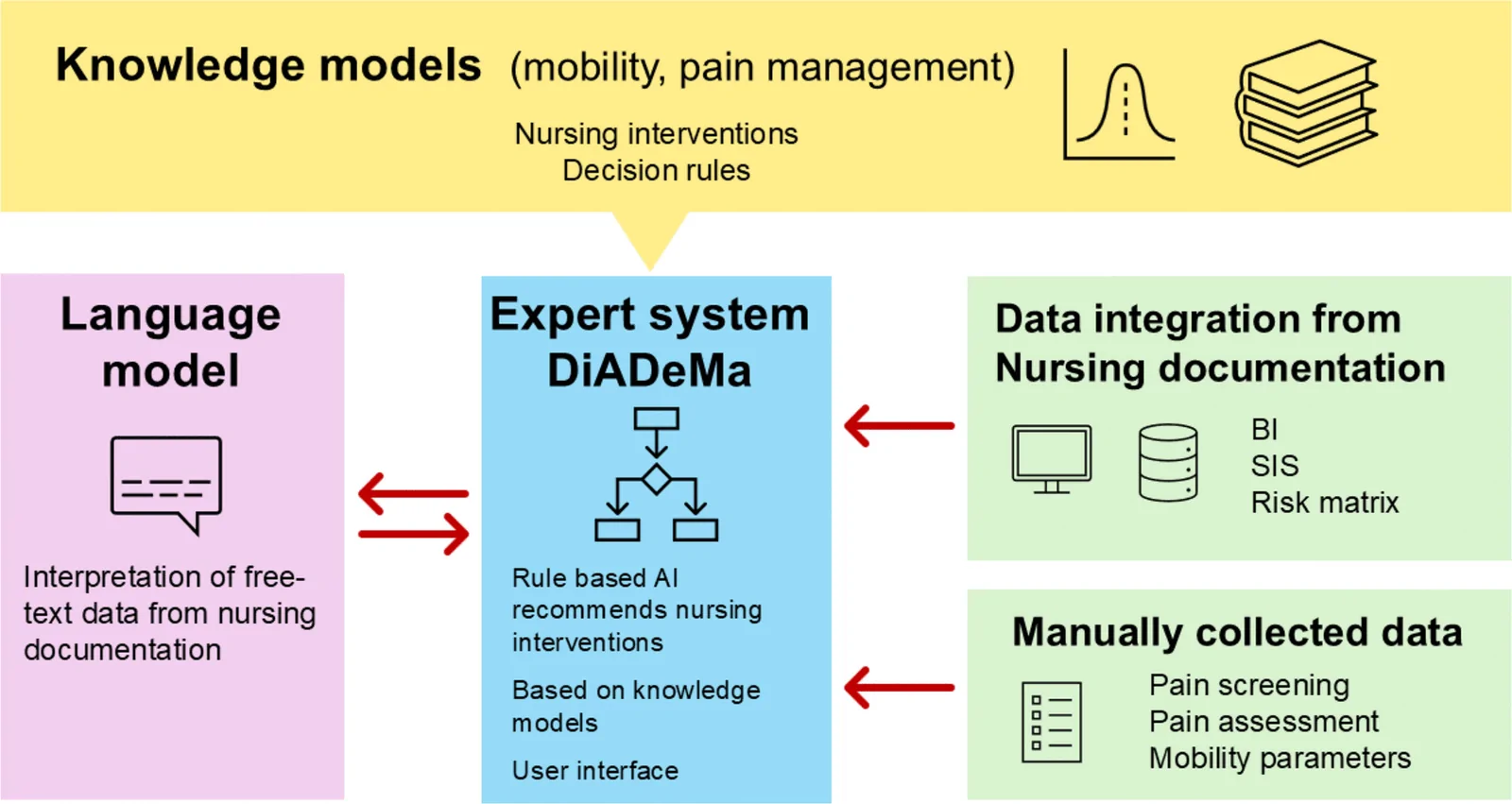
System overview with interacting components

The developed CDSS consists of three interacting components as shown in Fig. 3: (1) knowledge models, (2) expert system, and (3) language model, which will be described detailed in the following subsections. The development process followed an interdisciplinary co-design approach involving the nursing scientists, digitalization specialists, and data scientists, as well as practicing nursing professionals from partner nursing facilities as practice stakeholders. The co-design activities were designed to integrate discipline-specific expertise and to ensure feasibility, relevance, and usability [34, 35]. The co-design process was conducted in iterative cycles. Nursing scientists were responsible for the content of the knowledge models that map indicators in nursing documentation to the recommendation of nursing interventions. Digitalization specialists and data scientists worked on the technical development of the CDSS, the implementation of the rules from the knowledge models, and the training and deployment of the large language model that extracts relevant information from free-text. Furthermore, practicing nursing professionals from partner nursing facilities contributed by explicating current documentation workflows and available data sources, and by providing and contextualizing relevant nursing quality standards used in routine care. They further supported the validation of interventions derived from the literature and standards in terms of their practical relevance and assessed the interpretability and plausibility of the developed models.

Alternatives to a co-design approach could have been a participatory design focusing on user requirements or agile methods for continuous adjustments [36, 37]. We decided not to follow them because we wanted to focus on the CDSS architecture, specifically the inputs, outputs and methods and appropriate communication of the recommendations, not yet on the usability. The interdisciplinary co-design approach allowed us to combine various strengths of the different stakeholders.

Knowledge models were developed for each application area, reflecting the current state of the art in nursing care related to mobility and pain management. These models include decision rules for the recommendation of nursing interventions. The knowledge-based expert system DiADeMa (Digitally Assisted Decision Making) [38] serves as the technical core of the CDSS. DiADeMa employs a rule-based approach to generate nursing intervention recommendations. The decision rules derived from the knowledge models are implemented within the DiADeMa framework. Input data for the system are sourced from the nursing documentation, including the BI, SIS, and risk matrix. Additional input data that is not available in routine nursing documentation is collected manually (pain screening, pain assessment, additional mobility indicators). A language model is integrated into the system to facilitate the interpretation of free-text entries relevant to clinical decision-making in the application area mobility. The language model does not provide own nursing intervention suggestions but is only used to extract decision-relevant information from unstructured data. It retrieves data for decision making in DiADeMa.

### Knowledge models and decision rules

The system was developed in a generic form while focusing on the two exemplary and highly relevant application areas for nursing homes, mobility and pain management. A separate knowledge model was developed for each of the two application areas. They were developed by researchers of the nursing science partner of the project who hold degrees in nursing science and are registered nurses.

To develop the knowledge models, an initial analysis of the nursing care planning process was conducted. This analysis focused on (1) how information gathering is carried out and (2) which documents make up the nursing documentation, including their respective contents. In addition, a literature review was conducted to identify relevant nursing classifications, evidence-based publications, and applicable standards and clinical guidelines. Findings from the analysis of the nursing care planning process and the literature review were synthesized and used to inform the development of the knowledge models.

A list of interventions was generated for each area of application based on relevant literature (details are given in Sects. 3 .2.1 and 3.2.2 for mobility and pain management, respectively). The project’s practice partners were asked to revise and validate these interventions. Each intervention served as the starting point for the development of corresponding decision rules, which combined specific characteristics of one or more indicators. Thus, defining characteristics of nursing interventions were linked to the available data sources, relevant nursing interventions were selected, and decision rules were developed to build a recommendation framework for nursing interventions based on the documented data using an expert-driven approach. The development of the two knowledge models followed distinct approaches, reflecting differences in data availability and clinical documentation practices. The knowledge model for mobility was based on routinely documented nursing data, with only a limited amount of additional information collected. In contrast, for the pain management knowledge model, almost no suitable data to build knowledge models was available in routine documentation. Consequently, this model had to rely primarily on supplementary data collected within our project beyond routine documentation.

Testing and adjustment was an iterative process where the decision rules were tested by the research team. Based on testing, the knowledge models and rules were adjusted. Overall, for each knowledge model three iterations were ran.

#### Mobility

The knowledge model Mobility (knowMob) aims to represent common interventions to compensate residents’ impairments, (for example, transferring a resident from bed to the wheelchair, or positioning, either in full or partial assistance or guidance) and preventive interventions to promote mobility in older persons or to prevent falls. The 43 interventions provided in the knowMob should enable nurses to generate a realistic mobility-related nursing intervention plan.

As for the knowledge base, we draw on the following resources:

- The nursing classification ENP (European Nursing care Pathways) [39]. We selected 6 nursing diagnoses relevant to mobility in LTC (impaired bed mobility, impaired in the ability to maintain a seated position, impaired walking ability, impaired transfer ability, risk for impaired physical mobility, risk for falls) and used the interventions provided by the classification for these nursing diagnoses. ENP lists indicators for each diagnosis. We used these combinations as pivotal points to further develop the knowMob.
- The German National Expert Standard for maintaining and promoting mobility in nursing care [33]. We used the proposed risk factors for mobility impairment as indicators for knowMob. Additionally, the standard provides information on the evidence base for interventions to promote mobility.
- Studies on evidence-based interventions to promote mobility in older people in LTC. We included interventions that can realistically be implemented by nurses in LTC in German nursing homes. These are exergames (video games to promote physical activity [40–42]) and ADL-Training [43].
- Nursing care textbook of which we used an intervention with anecdotal evidence [44].

On that knowledge base, we generated a list of interventions that were then linked to indicators derived from the nursing diagnoses. Indicators were matched with available suitable data sources. For knowMob there are 41 indicators in total, serving as candidates for the knowledge model: 28 Items of the BI that originate from the BI-modules (1) mobility, (2) cognitive and communicative abilities, (3) behavior and psychological health, and (4) self-care. Although the BI is not used in direct patient care, it reflects the current need for nursing care of residents with sufficient accuracy and provides structured, standardized data. Additionally, we use 13 indicators that cannot be represented by the BI. These are six risk factors for immobility, three indicators related to fall prevention (fall risk, fear of falling, and falls within the last 12 months), the diagnoses Dementia and Epilepsy, use of visual aids, and use of mobility aids, such as wheelchair or wheeled walker.

**Fig. 4:**
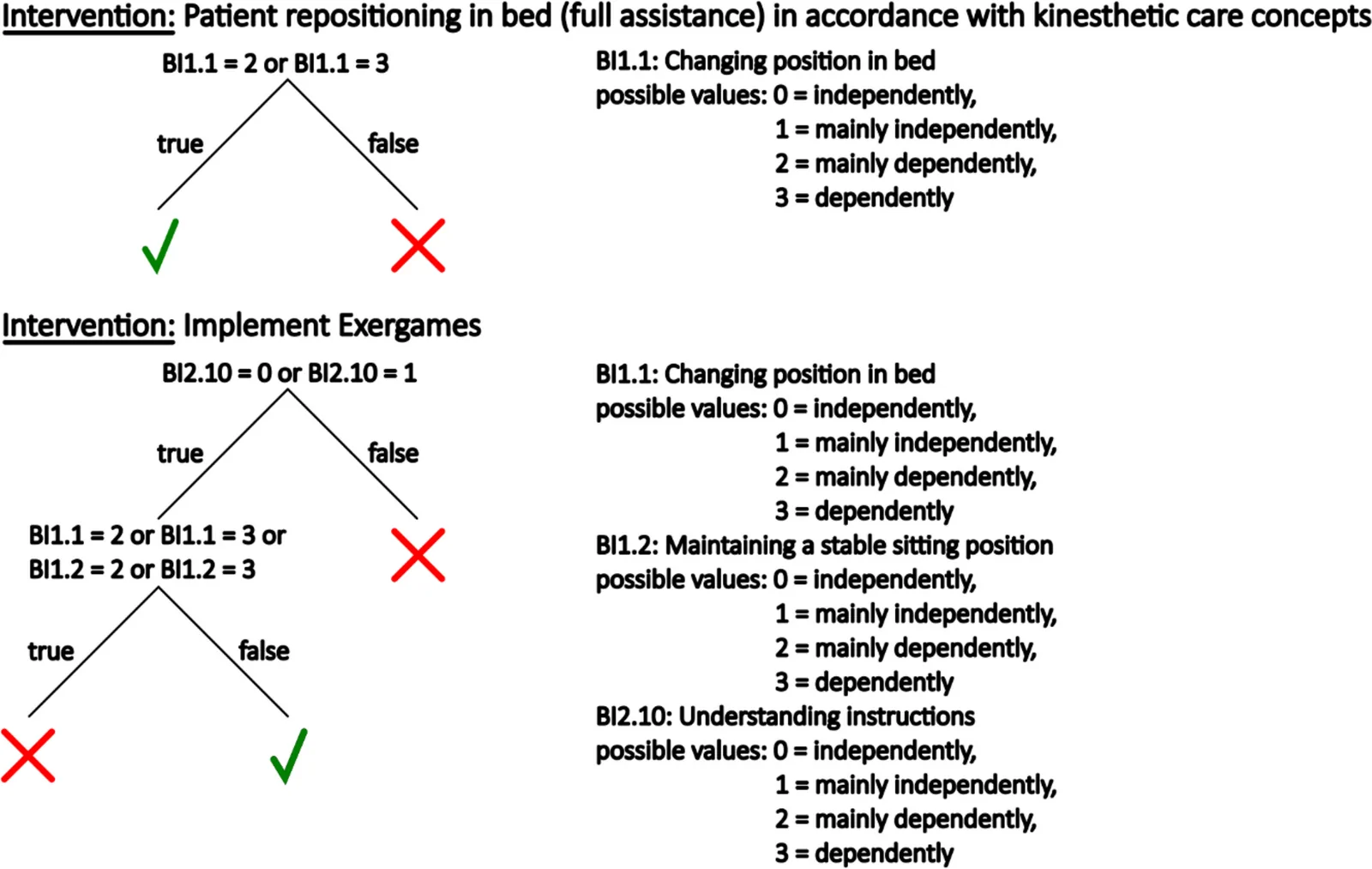
Two exemplary decision rules for mobility

The indicators are used as decision rules for each intervention, using 1 to 14 indicators (mode value: 1; median: 3). Examples for rules of different complexities are shown in Fig. 4.

#### Pain management

The knowledge model pain management (knowPain) aims at covering the process of assessing pain and pain treatment. Pain management is provided not only by nurses but also by other health professionals. Coordinating pain treatment by a multiprofessional team was therefore an important role as well. For the knowledge base we draw on the following resources:

- The Expert Standard for Pain Management in Nursing [31], the S3 Guideline: Pain Assessment in Older Adults in Long-Term Residential Care [32], and the S3 Guideline: Pain Management in Geriatric Patients across all Care Settings [45], which resulted from an update and expansion of the former S3 guideline All three aim to ensure that people in need of care receive effective and state-of-the-art pain management to prevent, reduce, or eliminate pain, improve quality of life, and support participation in daily activities.
- The Curriculum for Nursing Pain Management [46] that formulates competencies for nurses and aims at training nurses to provide professional pain management.
- Additionally, the classification ENP was consulted to cross-check interventions drawn from the standard and guideline. ENP, however, was of limited relevance, as standard and guidelines were more extensive sources.

The knowPain comprises 35 interventions in total, organized in four categories that represent the process of pain management:

- Interventions to identify and recognize pain
- Interventions for the assessment and systematic evaluation of pain progression
- Interventions to ensure coordinated pain treatment
- Nursing interventions for pain management and prevention

**Fig. 5:**
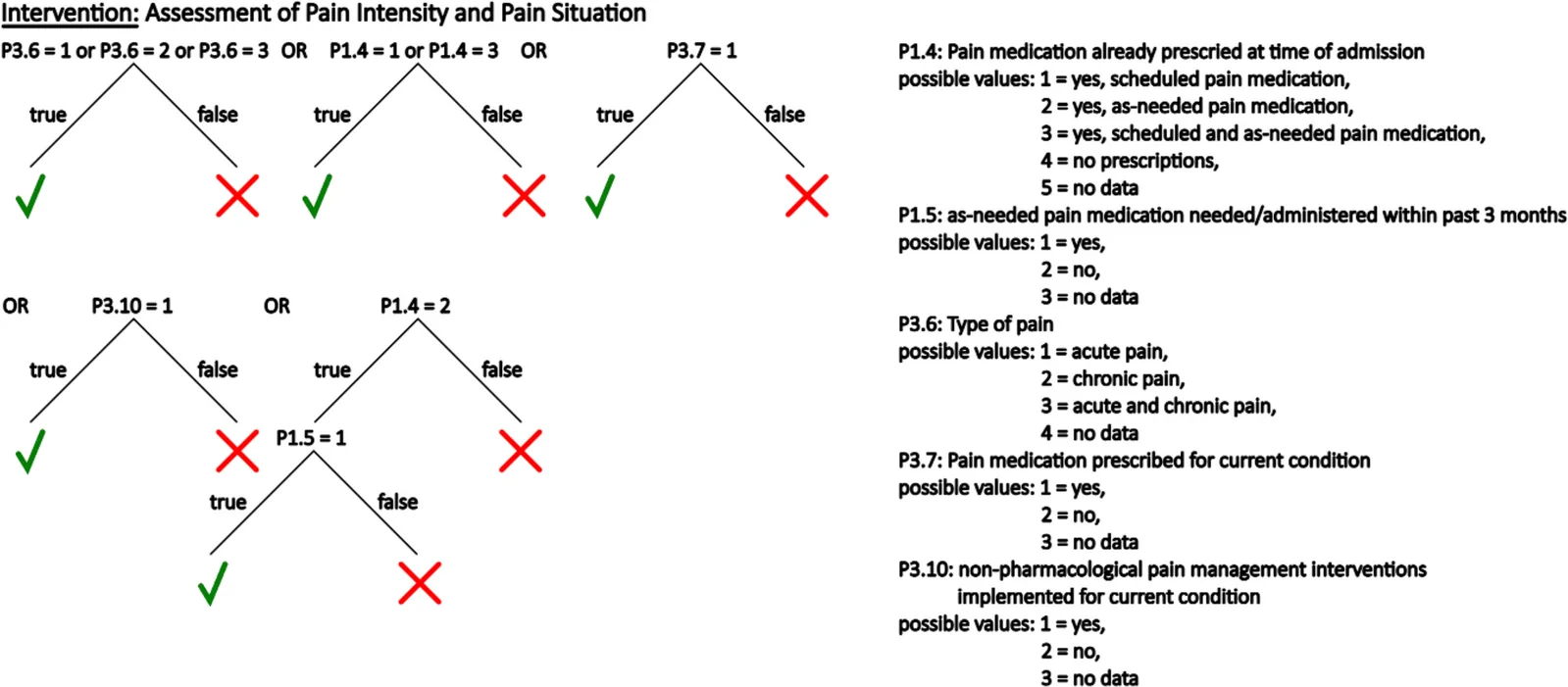
Example decision rule for pain management

Similar to knowMob, the interventions should be linked to indicators and respective data sources. However, there is almost no suitable data in the nursing documentation, especially the SIS. For developing knowPain and testing the prototype, we generated a list of indicators from the knowledge base (expert standard and guideline). During testing, these indicators were then collected manually by the nurses using the developed questionnaires. Data from the nursing documentation that can be included are BI items regarding the cognitive and communicative abilities of residents. The decision rules use 1 to 11 indicators (mode value: 1; median: 4). An example rule is shown in Fig. 5.

#### Summary

In total, 78 potential nursing interventions were identified to support comprehensive care planning across the two application areas: 43 for mobility and 35 for pain management. The decision rules were built as decision trees based on expert knowledge as described in the two previous sections. As input indicators, there are 46 possible indicators in the existing documentation, 13 additional indicators for mobility, and 21 additional indicators for pain management. Most of the rules use between two and ten indicators to decide whether a nursing intervention should be recommended or discouraged.

### Expert system

The decision rules were implemented in the CDSS DiADeMa, which consists of a C# backend and an Angular frontend, and which is configured with XML files providing information about indicators, interventions and decision rules.

DiADeMa supports parameters of several types, such as Boolean, Integer, Float, Date, Ordinal, or Nominal. The rules are encoded in a both human-readable and digitally processible language syntax based on 2nd order predicate logic. This syntax comprises operators of different type, such as elementary logic (NOT, AND, OR, EQUAL, …), arithmetic (PLUS, MINUS; MULTIPLY; DIVIDE, MIN, MAX, COUNT), or advanced logic (ANY … FULFILLS, ALL … FULFILLS). Exemplary rules have been presented in Sect. 3.

**Fig. 6:**
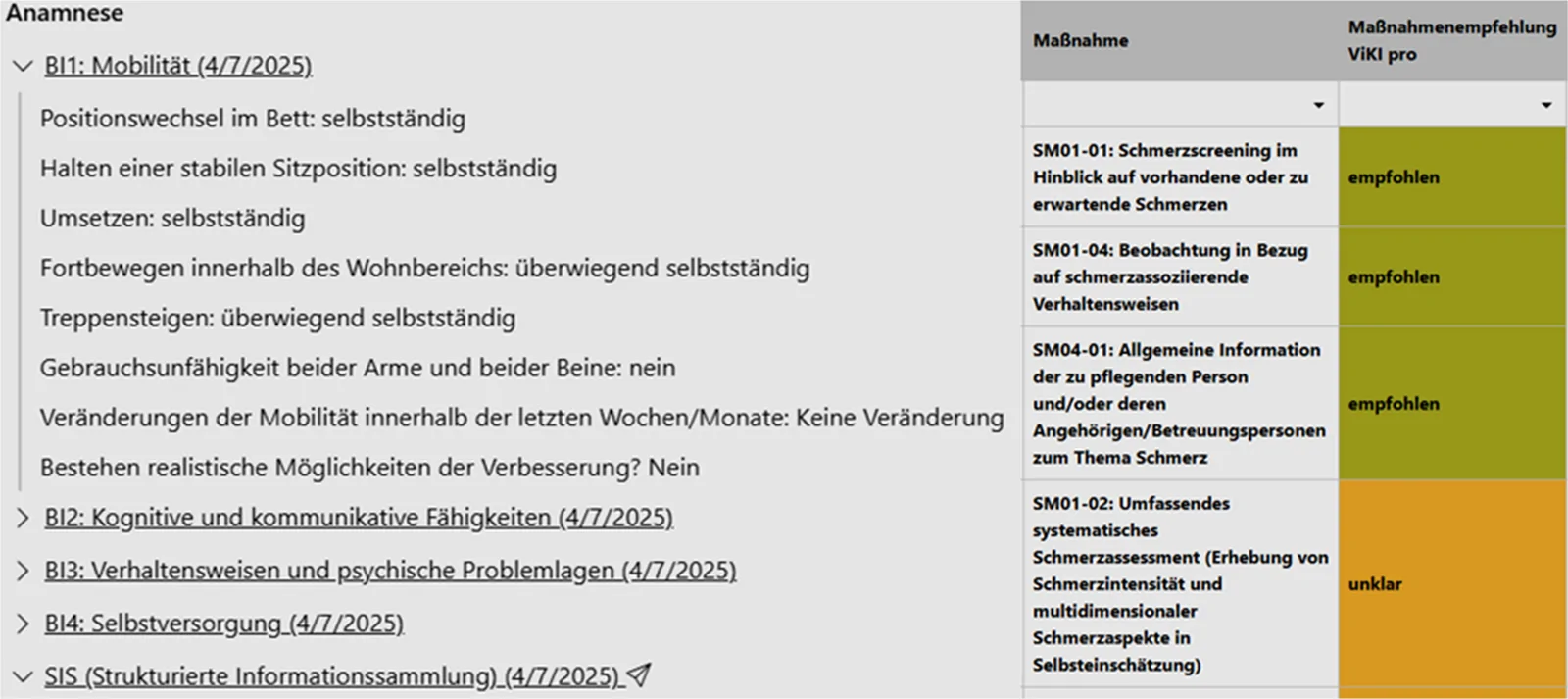
Extract of anamnesis (left part) and recommendations (right part – left column shows nursing interventions and right column shows recommendation degree where “empfohlen” = “recommended” and “unklar” = “unclear”) in DiADeMa (screenshot of original German version)

Figure 6 shows an example case in DiADeMa with some of the indicators that are used in the decision rules (left part) and the upper part of the recommended nursing interventions (right part).

### Large language model

To support the structured interpretation of free-text care documentation, a transformer-based large language model (Llama 3.1, 8B parameters) was locally deployed. The model was not further trained or fine-tuned in this study. Instead, it was applied using prompt engineering in a zero-shot configuration [47], meaning that no task-specific examples or training were provided and the model relied solely on natural language instructions to extract care-relevant indicators. Llama 3.1 follows a standard transformer-based decoder-only architecture as described in the original model documentation. The term “8B parameters” denotes the approximate number of about 8 billion trainable parameters of the model.

The model receives unfiltered narrative entries from the SIS and is prompted in natural language (German) to extract or classify care-relevant indicators for the mobility knowledge model. The large language model itself does not provide nursing intervention suggestions but is only used to extract relevant information from the unstructured SIS. It operates on a set of predefined mobility-related indicators (see Sect. 2.1), which are conceptually organized into two higher-level dimensions: *general medical history* and *mobility and daily life indicators.*

Two task types are distinguished: (1) classification tasks with categorical outputs (e.g., Yes/No/No indication), and (2) extraction tasks targeting the identification of specific terms (e.g., “rollator”, “glasses”). Both task types require the model to provide verbatim textual evidence from the original documentation to support its output. Outputs are returned in structured JSON format for downstream processing.

The language model was developed in Python and deployed via an Ollama Docker container running on a high-performance laptop equipped with an NVIDIA RTX 4090 GPU. During testing, the entire inference process was executed locally and offline, ensuring GDPR-compliant data handling within the care facility's internal network.

## Evaluation

### Testing procedure

A first exploratory testing of the CDSS was conducted in two care facilities in Cologne, Germany, in March and April 2025 for one week per care facility, which feature the characteristics as shown in Table 2.

**Table 2:** Characteristics of the participating facilities

Characteristic	Facility 1	Facility 2
Number of staff (n)	100	78
Number of residents (n)	75	80
Digital infrastructure	Electronic nursing documentation system	Electronic nursing documentation system
Quality development activities	Quality management; practice instruction/mentorship; participation in external quality audits	Quality management; practice instruction/mentorship; participation in external quality audits

The size of the participating facilities corresponds to the national German average of 79 places per facility [48]. The number of staff depends on the care level of the residents [49]. This is also reflected in the two participating facilities: In Facility 1, residents are cared for from care dependency level two to five, whereas Facility 2 also provides care for individuals without an assigned care level. Both facilities actively use instruments for quality assurance in nursing care.

Care-related information from 11 residents, five from the first care facility and six from the second, was used to evaluate the system’s decision-making performance. The cases used for the evaluation were independent of those used during prototype development. For each resident, two raters independently rated the suggestions generated by the system, resulting in 22 tests. In total, eight raters were conducting the tests in the two nursing homes. All raters were registered nurses. None of the raters is an author of this manuscript.

The relevant indicators (see Sect. 2) were either derived from the existing documentation or when necessary, additionally collected by a registered nurse and transferred into the system. The evaluation encompassed the decision rules and the language model. The procedure consisted of the following steps, which were performed in a single session for each resident.

First, the indicators of the BI and of the SIS were transferred into the CDSS. While data could be transferred automatically in the second care facility, it had to be manually filled in in the first one due to limitations in the local IT infrastructure.

Second, the additional indicators for mobility management were filled in by the language model. The proposals of the language model were rated, and errors were corrected manually to have complete documentation for the nursing intervention rule base. The rater had the option to completely agree with the proposal of the language model, to agree with the proposed value (e.g. “glasses” for visual aid) without agreeing with the language model’s explanation, or to reject the language model proposal. These ratings were part of the 22 tests described above with approximately ten ratings per test.

Third, based on the predefined decision logic, the system labeled all 78 nursing interventions either as “recommended”, “unclear”, or “discouraged”. Here, “unclear” means that the documented values do not allow a clear suggestion for or against a nursing intervention. The rater was asked two questions for each nursing intervention:

1. Is the suggestion of the CDSS, i.e. the labeling as “recommended”, “unclear” or “discouraged”, correct?
2. Would you transfer this nursing intervention into your care plan?

The first question should evaluate the correctness of the knowledge model. In absence of a gold-standard, this was evaluated indirectly by asking nursing professionals whether the suggestion generated by the rules was appropriate for the individual care situation. The second question should detect potentially irrelevant nursing interventions or potential gaps in the standard care plan of the care facilities.

The collected data was analyzed using the statistical software R (version 4.4.1) [50] and Python (version 3.9).

### Results

All results shown in this section are only of descriptive nature due to the small sample size, the clustered data structure (many ratings by only few persons), and the fact that cognitive-load effects during testing may have biased the ratings.

#### Expert-rated knowledge model correctness

**Fig. 7:**
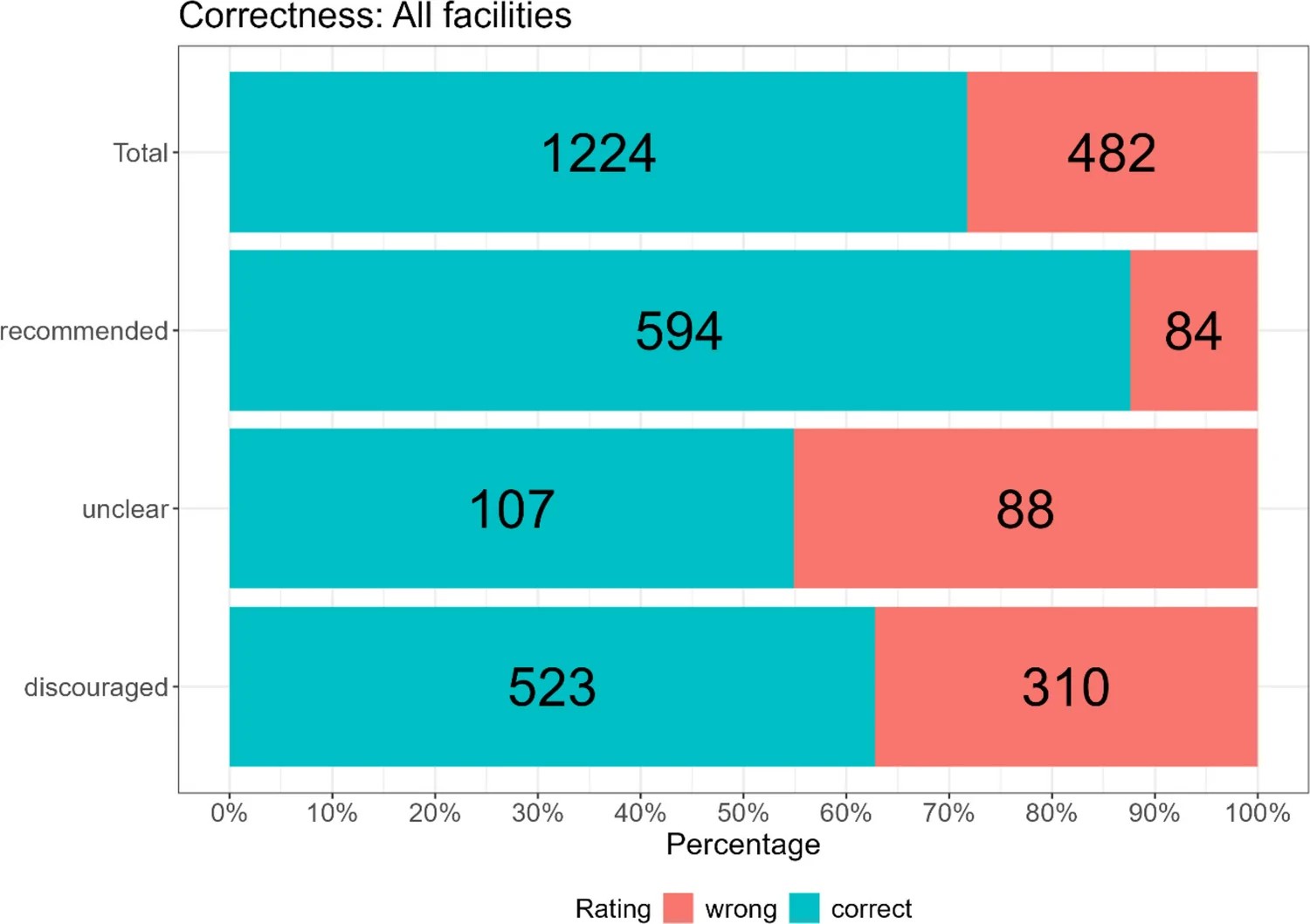
Total results of correctness analysis

The first question treated the correctness of the decision rules and was evaluated by expert ratings. In total, 1,706 ratings were made and 10 suggestions were not rated. Among them, 1,224 (72%) were “correct” and 482 (28%) were “wrong”. Under the 678 “recommended” suggestions, 594 (88%) were deemed “correct”, and 523 (63%) of the 833 “discouraged” suggestions were rated as “correct”. These results are shown in Fig. 7. An “unclear” suggestion was rated as “correct” if the available information in the patient case does not allow a decision of whether the corresponding intervention can be recommended. There were two raters per case. In 67% of the suggestions, these two raters agreed (83% in “recommended” suggestions, 34% in “unclear” suggestions, and 61% in “discouraged” suggestions).

**Fig. 8:**
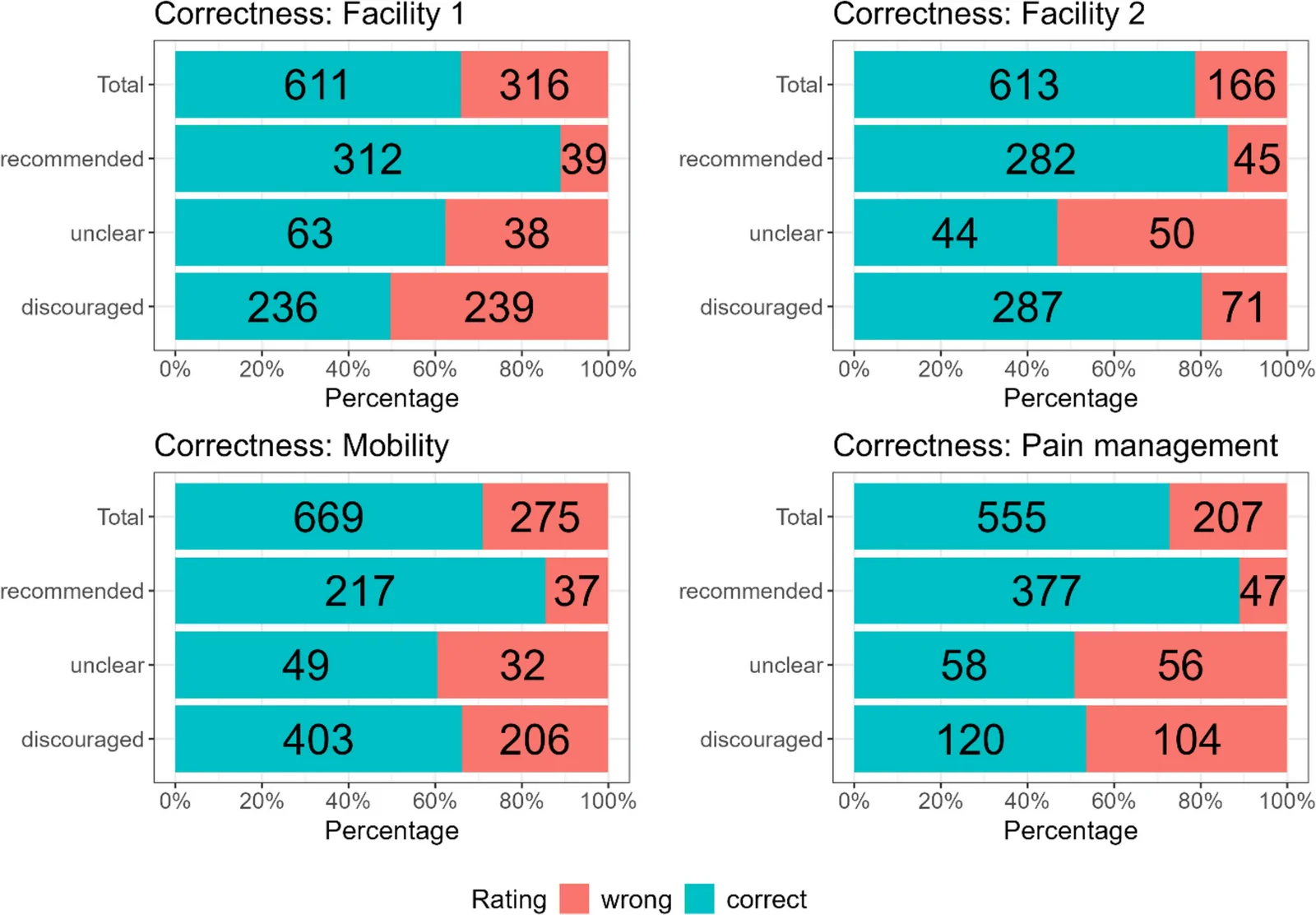
Results of correctness analysis split by facility and by application field

Figure 8 presents detailed results split by facility and application area. When splitting between care facilities, we observe much higher “correct” rating rates in Facility 2 (79%), where the system was evaluated at a later point of time than in Facility 1 (66%). This observation is particularly expressed among the “discouraged” suggestions (50% “correct” in Facility 1, 80% “correct” in Facility 2).

For mobility and pain management, the expert judgement on the system showed very similar results. While 669 of 944 (71%) mobility suggestions are rated as “correct”, these numbers amount to 555 of 762 (73%) suggestions for pain management.

#### Transfer to care planning

The second question was about the transfer of the recommended nursing interventions into real-world care planning. Here, the focus is not on the overall rate of transferred interventions. Instead, it is desired that many of the recommended nursing interventions are transferred to care planning while few of the discouraged nursing interventions should be transferred. There are several reasons why recommended nursing interventions may not be transferred to care planning. First, the recommendation may be incorrect (see Sect. 3.3). Second, two or more nursing interventions may address the same issue, making some of them redundant. Finally, some interventions may be suitable but are not applied in the specific care facility.

**Fig. 9:**
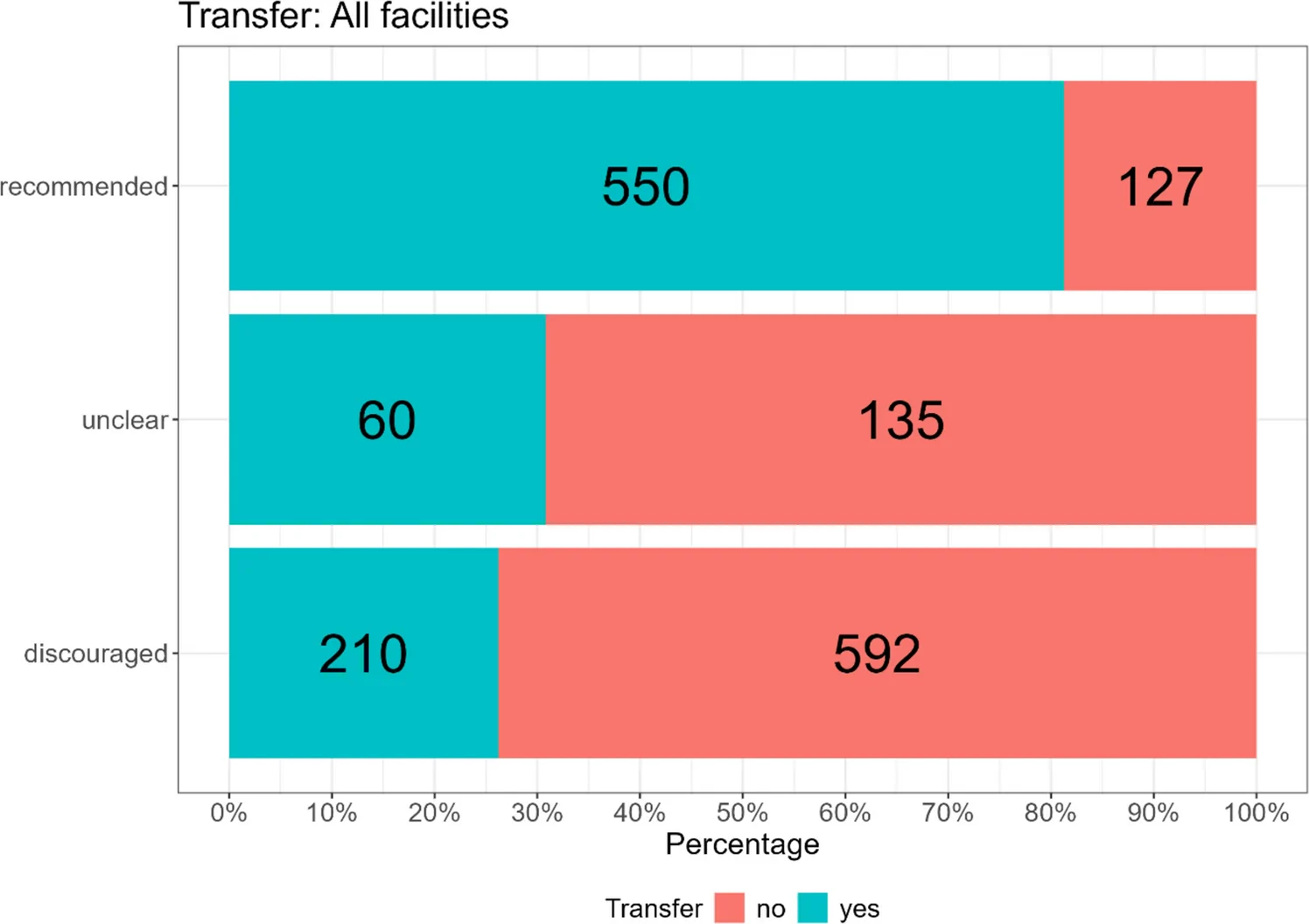
Total results of transfer analysis

Figure 9 shows the results for the entire test. Following our evaluation, 81% (550 out of 677) of the recommended nursing interventions would be transferred to intervention planning. Among the “discouraged” suggestions, 26% (210 out of 802) nursing interventions would be transferred to intervention planning.

There were two raters per case. In 76% of the suggestions, these two raters agreed (78% in “recommended” suggestions, 65% in “unclear” suggestions, and 77% in “discouraged” suggestions).

**Fig. 10:**
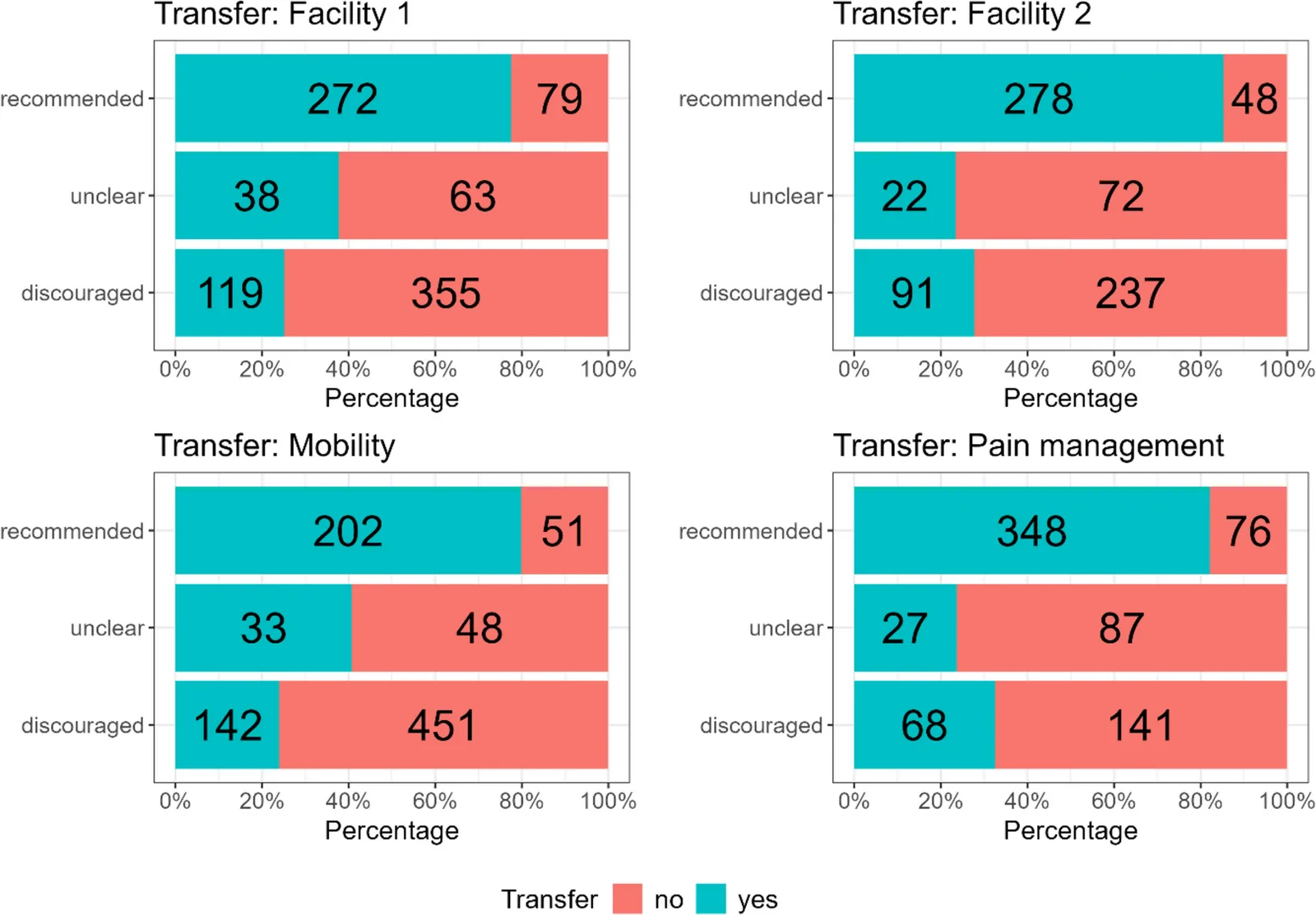
Results of transfer analysis split by facility and by application field

The results can be split by facility and application field. The results are shown in Fig. 10. Again, in the second facility, slightly better results are observed compared to the first facility. Among the recommended nursing interventions, 77% (272 out of 351) would be transferred to intervention planning in Facility 1 and 85% (278 out of 326) in Facility 2. Regarding the discouraged interventions, the values are more similar (25% in Facility 1 and 28% in Facility 2).

The results for the application fields mobility and pain management are once again more similar. 80% (202 out of 253) recommended mobility nursing interventions would be transferred to care planning compared with a value of 82% (348 out of 424) for pain management. Among the discouraged nursing interventions, these values amount to 24% for mobility and 33% for pain management. This indicates a slightly higher transfer rate for pain management.

After rating all nursing interventions, the raters were asked if they could design a correct nursing plan with this system. They were asked for each rating, so the same registered nurse may have answered multiple times, as the answer may vary depending on the specific care situation. For mobility, 67% raters (12/18) answered that this is entirely possible and 33% (6/18) said that it is largely possible. For pain management, 60% (12/20) said that it is entirely possible to design a correct nursing plan. 35% (7/20) said that this is largely possible and 5% (1/20) answered that it is rather not possible. Furthermore, 24% (4/17) answered that they learned nursing interventions that they have not known before and 24% (4/17) answered that the system contains nursing interventions that are not applied in their facility.

#### Large language model

The locally deployed LLM functioned in this implementation as a semantic interface between unstructured nursing documentation and the expert system.

During the evaluation period, 219 narrative documentation entries were processed automatically. The model outputs always included, in addition to the indicator assignment, verbatim excerpts from the original documentation, ensuring transparent traceability for subsequent expert review.

**Fig. 11:**
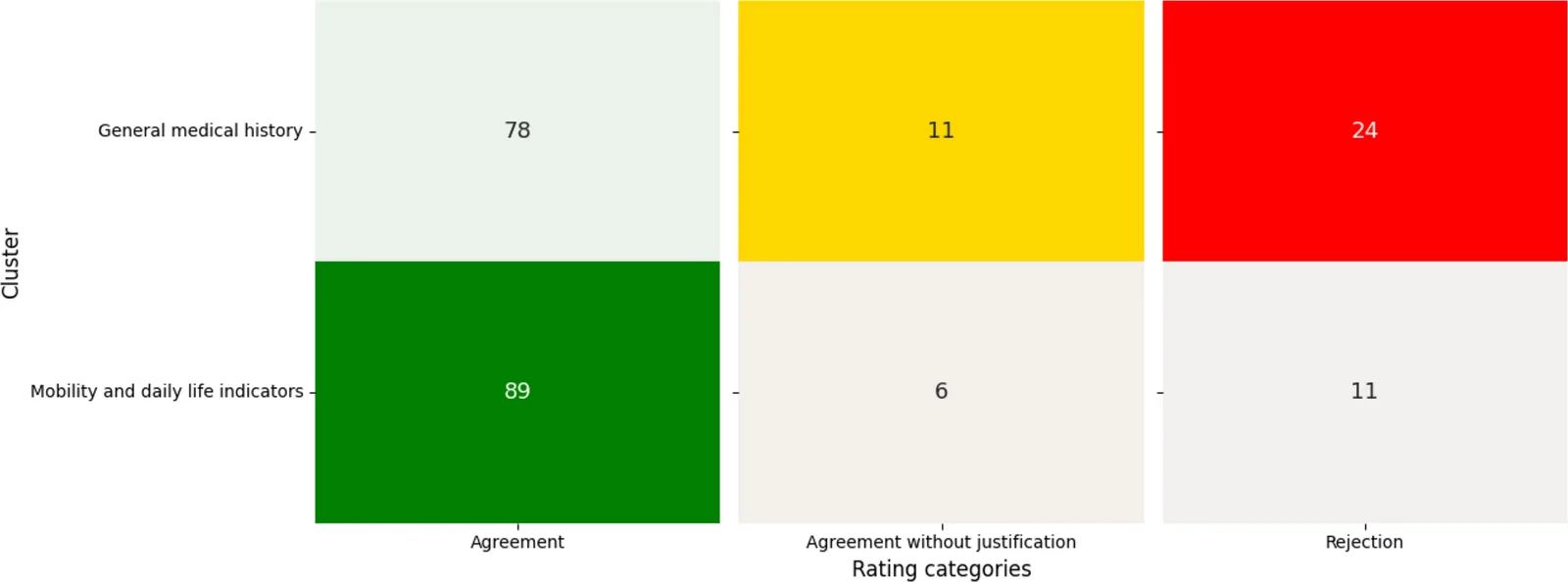
Distribution of expert judgements for the two indicator clusters (General medical history and Mobility and daily life indicators). Agreement levels were higher for mobility‑related and daily life indicators (89 of 106 (84%) full agreement) compared to general medical history indicators (78 of 113 (69%) full agreement)

For the analysis, the 12 indicators were grouped into two thematic clusters: (1) **general medical history** (113 entries) and (2) **mobility and daily life indicators** (106 entries) (see Sect. 2.1). The results show that the LLM produced notably more consistent and comprehensible assignments in Cluster 2 (see Fig. 11). In this cluster, full agreement was 84% (89/106), “agreement without justification” accounted for 6% (6/106), and rejections were only 10% (11/106).

In Cluster 1 (General medical history), by contrast, only 69% (78/113) of suggestions were fully agreed with, 10% (11/113) were rated as “agreement without justification”, and 21% (24/113) were rejected. The higher divergence rate in this cluster was mainly due to missing statements or ambiguous wording in the primary documentation, which made unambiguous indicator assignment more difficult. The LLM could still generate plausible suggestions. However, these required expert correction more often.

Overall, this yields a fully agreement rate of 76% (167/219). The findings highlight that the LLM handles mobility‑related features with high reliability, but complex anamnesis‑related information remains an area where expert oversight is essential during the evaluation.

## Discussion

In this paper, we presented an expert-knowledge-driven approach for developing a CDSS for care planning in nursing homes. Furthermore, it was demonstrated how LLMs can assist in extracting relevant information from free-text documentation. The approach is based on data sources available in Germany, but the overall strategy is applicable to other countries as well. An initial testing period in two care facilities with a total of 11 care cases, each tested twice (22 test procedures), provided insights into the current quality of the CDSS’s suggestions.

### Testing procedure and results interpretation

An important finding of the exploratory analysis is that 72% of the system’s suggestions were rated as “correct” by nursing professionals. We expect that this rate can be improved by revising the rule base, more precise formulations of the questions to be answered by the raters, and by implementing a better pre-testing process (see the detailed discussion below). However, it is not trivial to define an optimal target rate. A “correct” rate of 100% might imply overfitting to the few raters and the two care facilities. Given the individualized character of planning nursing interventions and the absence of a gold-standard that defines correctness, values below 100% might be the better target in similar frameworks.

When evaluating which of the suggestions were “correct” or “wrong”, we observe a much higher “correct” rate among the “recommended” nursing interventions (88%) compared to the “discouraged” ones (63%). This may be caused by a misconception, as a “discouraged” suggestion can be either correct or incorrect. We suppose that the lower approval rate may be partially linked to misunderstandings arising from the double-negative logic in interpreting a non-recommendation. When evaluating 78 nursing interventions and answering two questions for each, it is conceivable that a rater, due to lapses in attention, may mistakenly rate a correct “discouraged” suggestion as “wrong”, especially if the intervention seems wrong at first sight. Although only the system's suggestion was intended to be evaluated, some raters may have focused at times on the correctness of the intervention itself. To prevent such misunderstandings in future studies, we recommend using more precise wording. Furthermore, rater feedback indicated that performing data entry and evaluation in one step required a high level of concentration. While a two-step procedure can help maintain concentration and reduce cognitive load, it may be more difficult to implement in practice within care facilities. Such trade-offs should be considered when designing future testing procedures.

The consistently better ratings in the second care facility support the hypotheses formulated in the previous paragraph. While the CDSS itself, including the knowledge models, decision rules, and LLM configuration, remained unchanged between both evaluation periods, there were differences in procedural aspects. First, there was a learning effect in presenting the testing procedure between the two testing periods on the part of the researchers. In particular, the issue of mistakenly incorrect ratings of the “discouraged” suggestions was highlighted based on insights gained in the test procedure in the first facility. Moreover, in the second facility, most of the data were imported directly from the electronic care documentation system, which significantly reduced the time required for manual data entry and consequently, the overall time and level of attention needed. The interface enabling this data transfer could only be implemented in the second facility, as the first facility experienced technical problems with data transfer that were unrelated to the CDSS.

These observations underline the importance of pre-testing, not only to validate technical functionality, but also to identify potential sources of misunderstanding and cognitive overload. We therefore consider a structured pre-test period that takes these aspects into account to be essential for future studies.

At a broader level, the findings regarding the integration of CDSS into electronic health and care documentation systems align with those of Akbar et al. [24]. They found in their systematic review that integrating CDSS into EHRs results in better outcomes compared to standalone tools. They emphasize that automation reduces information gaps, prevents data duplication, and enhances usability—benefits that were mirrored in our own study through the streamlined data flow and reduced participant burden enabled by system integration in the second facility.

### Large language models to access unstructured nursing data

The use of LLMs to analyze unstructured nursing documentation offers considerable potential for decision support in long-term inpatient care. Unlike structured input forms, narrative documentation often contains nuanced observations and contextual details that are essential for individualized care planning. LLMs enable the systematic extraction of such information from free-text entries, even when expressed variably or only implicitly.

Our evaluation showed that model-generated suggestions aligned well with expert ratings for clearly observable and routinely documented indicators, such as movement limited to one’s own room or visits from relatives. In contrast, performance was weaker for more complex, amnesia-related indicators, such as dementia or epilepsy. This discrepancy might be primarily because such information is often not explicitly mentioned in nursing notes. Instead, it may be expressed implicitly or stored in other systems, such as medical records. In the zero-shot setting without additional training or context integration used here, the model lacks the necessary background to make valid judgments in these cases. To improve performance on such complex indicators, a broader data foundation may be required. This could include access to supplementary data such as diagnoses or care reports, integration across multiple documentation systems, or semantic enrichment. While fine-tuning LLMs on domain-specific data is another possible strategy, it typically requires large, annotated datasets, which are rarely available in routine nursing care.

Another consideration is the technical deployment of LLMs in care settings. Local hosting via containerized environments, for instance, helps meet data protection requirements by preventing sensitive data from being transferred to external cloud services. However, this approach requires substantial computing resources, particularly dedicated GPUs, and advanced infrastructure, both of which are typically unavailable in most care facilities. Consequently, the local operation of LLMs at scale is currently not feasible in these settings. In addition to the high initial investment required, the ongoing need for technical maintenance and administrative support presents further barriers to sustainable implementation. While the models themselves are becoming more accessible, their operational requirements remain incompatible with the technical environment of most care institutions.

### Data collection in nursing care

The CDSS was initially developed to support care planning following a resident’s admission. The analysis of the data sources available at the beginning of the care process, using the application areas mobility and pain management as examples, revealed several key challenges for the development of a rule-based system regarding data availability:

- The necessary information was not fully contained in any single document, nor was it reliably represented across all available records.
- In many cases, the data were unstructured or inconsistently formatted, which significantly hindered automated processing.
- Additionally, relevant information was often scattered across different sections of the documentation or labeled in ambiguous ways.

It is conceivable that only care-relevant information, such as risks and limitations, that is meaningful in the given case is documented, and that the absence of certain data merely reflects their lack of relevance for the individual case. Based on the available literature [29, 30] and documentation, however, it is unclear which documentation principle is applied in the nursing records examined here.

These findings indicate that routine care data in Germany can only serve as a limited foundation for the development of a broadly applicable, rule-based decision support system. To extend the system’s use beyond the initial admission phase, it is essential to incorporate information from the ongoing care process, particularly to enable the continuous adjustment of nursing interventions. A central data source in this context is the nursing report, which, in the German care context, like the SIS, is still predominantly documented in free-text format. This aligns with findings from the Netherlands, where up to 80% of data in EHRs are unstructured, free-text, and narrative data, resulting in similar challenges regarding usability in CDSS [39].

The use of standardized nursing language and nursing terminologies, taxonomies, and classifications in digital systems is essential for effectively integrating digital applications into nursing practice and for representing nursing processes in a structured and transparent manner [23, 51, 52]. To ensure interoperability, ontologies should be further developed and used in nursing [51–53]. When such standardized language is not employed, digital nursing documentation systems, electronic care planning tools, and computerized decision support systems face substantial challenges. To address poor data quality and completeness Nursing Minimal Data Sets (NMDS) are discussed in the literature [54–56]. NMDS are standardized sets of nursing data collected on a regular basis in a standardized and comparable format. They can ensure both data completeness and quality and can be seen as a basis for further development of CDSS in nursing care.

### Limitations

The presented work has several limitations. First, the CDSS and the underlying rule base were developed based on the German nursing documentation, professional nursing standards, and evidence-based literature mainly used in German-speaking countries. Therefore, the developed knowledge models should be interpreted within this context. The general methodology for developing a CDSS in long-term care, however, could be applicable to other countries as well but remains to be validated there.

Second, eight raters evaluated the system on 11 cases, with two ratings per case, resulting in 22 ratings in total. This sample size only allows for exploratory analysis and, therefore, the results are of descriptive nature.

Third, inter-rater agreement was not achieved in all cases. As no clear established gold standard for either the evaluated nursing interventions or the underlying large language model’s suggestions was available, the assessment relied on expert judgment. As a result, classical quantitative evaluation metrics for the LLM, such as precision or recall, could not be meaningfully applied.

Despite these context-specific aspects, the overall methodology, including the model development process, the integration of a LLM, and the evaluation procedure, may be of interest to researchers in other countries and can be adapted to different documentation frameworks and clinical contexts.

### Directions for future work

The first version of a prototype for a CDSS for nursing interventions presented here opens numerous directions for future research. As described in Sect. 4.2.2, the developed CDSS is currently only applicable to German nursing homes as the underlying knowledge model relies on the German nursing data. The presented methodology could be applied to other regions as well to develop a similar knowledge base.

The results of information extraction from unstructured text using an LLM were promising in the application field “mobility”. A next step could be to collect plain texts for “pain management” as well and to run the LLM regularly on the continuously written nursing report to extract information that is relevant for decision making. Such a procedure requires digitalization of all documents in the nursing home, integration of an LLM into the care documentation system, and sufficient computing power on the care facilities’ machines. Addressing all these challenges might result in an interesting and beneficial project.

In this project, a total of 78 nursing interventions were provided to the registered nurses for the two application fields mobility and pain management. Developing a knowledge model to capture the nuanced differences between these interventions poses a significant challenge. Furthermore, it may be time-consuming for the registered nurses to consider many similar nursing interventions on an equal recommendation level. An accompanying data-driven approach, considering similar cases and which nursing interventions were successful for them, could help address this issue. Here, recommendation levels could be divided more finely, for instance, by their alignment with other cases. Creating such an approach requires a sufficiently large database containing care cases and success ratings of implemented nursing interventions. The collection of such a database and the development of the data-driven recommendation system would be a promising topic for future research.

The points outlined in Sect. 4.2.1, combined with the absence of standardized nursing language present major barriers to structured data analysis. As a result, critical information may either be missing from the routine data or may not be reliably identified or interpreted by the system. Consequently, the rule base cannot be comprehensively modeled, at least not in a way that enables decision support across the full range of care situations. This underscores the need for more structured documentation practices and a clear semantic modeling of care-relevant content to ensure that such information is machine-readable and unambiguously interpretable within rule-based systems. It also raises the broader question of how nursing documentation can be aligned with both the requirement for individualized care planning and the need for standardized, technology-supported decision-making without losing the ability to capture the complexity and individuality of various care situations. This question opens up an interesting field for future research.

## Data Availability

Data cannot be shared to protect study participant privacy.
